# The Role of Compost in Stabilizing the Microbiological and Biochemical Properties of Zinc-Stressed Soil

**DOI:** 10.1007/s11270-017-3539-6

**Published:** 2017-08-24

**Authors:** Rafał Strachel, Jadwiga Wyszkowska, Małgorzata Baćmaga

**Affiliations:** Department of Microbiology, University of Warmia and Mazury in Olsztyn, Plac Łódzki 3, 10-727 Olsztyn, Poland

**Keywords:** Zinc, Soil, Contamination, Compost, Biostimulation, Microorganisms and enzymes

## Abstract

The progressive development of civilization and intensive industrialization has contributed to the global pollution of the natural environment by heavy metals, especially the soil. Degraded soils generally contain less organic matter, and thus, their homeostasis is more often disturbed, which in turn manifests in changes in biological and physicochemical properties of the soil. Therefore, new possibilities and solutions for possible neutralization of these contaminations are sought, inter alia, through reclamation of degraded land. At present, the use of additives supporting the reclamation process that exhibit heavy metal-sorbing properties is becoming increasingly important in soil recovery. Research was conducted to determine the role of compost in stabilizing the microbial and biochemical balance of the soil due to the significant problem of heavy metal-contaminated areas. The study was conducted on loamy sand, to which zinc was applied at the following doses: 0, 250, 500, 750, 1000, and 1250 mg Zn^2+^ kg^−1^ DM of soil. Compost was introduced to the appropriate objects calculated on the basis of organic carbon content in the amount of 0, 10, and 20 g C_org_ kg^−1^ DM of soil. The study was conducted over a period of 20 weeks, maintaining soil moisture at 50% capillary water capacity. Zinc significantly modified soil microbiome status. The abundance of microorganisms and their biological diversity and the enzymatic activity of the soil were affected. The negative effects of contaminating zinc doses were alleviated by the introduction of compost into the soil. Organic fertilization led to microbial growth intensification and increased biochemical activity of the soil already 2 weeks after compost application. These effects persisted throughout the experiment. Therefore, it can be stated that the use of compost is an appropriate method for restoring normal functions of soil ecosystems contaminated with zinc.

## Introduction

The intense growth of the human population entails the need to increase agricultural production and crop area. Therefore, modern agriculture is facing the great challenge of providing sufficient quantity and quality of food for humanity. In turn, the increase of urbanization and the diversity and growth dynamics of contaminations present in the soil environment endanger both crop yields and their wholesomeness. Particular attention is paid to the heavy metal contents of the soil due to their persistence in the environment, as evidenced by works on the role of trace elements in the soil environment and their penetration into the trophic chain (Verma and Dwivedi 2013; Vrščaj et al. 2008; Wyszkowska et al. 2013). Some of these elements are essential for the correct growth of plants and the functioning of complex systems of biological processes that could not exist without their presence (Chibuike and Obiora 2014). Others do not affect the functioning of living organisms and the course of biological processes occurring in the soil environment, thus their occurrence in the soil may be undesirable. Zinc belongs to the elements involved in various biological processes. However, from an ecological point of view, excessive amounts of this element result in a number of adverse effects (Wyszkowska et al. 2013; Wyszkowska et al. 2017; Strachel et al. 2017a; Strachel et al. 2017b).

Crop yield is dependent on soil fertility, which in turn is determined by the microbiological and biochemical properties of the soil, which are closely interrelated. The state of the soil microbiome, both in terms of quantity and diversity, influences the activity of soil enzymes. The decomposition of organic matter, and hence the release of nutrients is the result of enzyme involvement in these processes (Chaperon and Sauvé 2007; Kızılkaya et al. 2004; Utobo and Tewari 2015). Therefore, it is very important for modern agriculture to maintain a high biochemical activity of the soil. Zinc in moderate amounts contributes to the stimulation of soil microbiome and its activity, while in excessive doses it disturbs soil homeostasis. The abundance and diversity of soil microorganisms is disrupted, which can also contribute to the deterioration of soil biochemical properties (Boros et al. 2011; Wyszkowska et al. 2016). Bioavailability of these elements is the most important aspect of heavy metal pressure in the soil. The total quantity of heavy metal may remain unchanged but its toxicity is dependent on its unbound form (Luo et al. 2012). This balance is determined by specific soil environment conditions, i.e., pH, organic matter content, and sorption complex volume (Aydinalp and Marinova 2003). Soils heavily degraded by heavy metals need a sufficient amount of stabilized organic matter, which not only delivers nutrients and reduces their losses, but also shows the ability to bind these elements. Replenishment of organic matter in contaminated soil can be achieved through the application of compost, which effectively increases soil fertility. Compost supplies vast quantities of nutrients to the soil, which become more available and assimilable to soil organisms. In addition, it improves soil physicochemical properties such as soil density, soil porosity, water and heat capacity, soil sorption and aeration (Adugna 2016; Babalola et al. 2012; Civeira 2010; Pane et al. 2015). The presence of organic matter in the compost also contributes to the promotion of microorganisms in the soil environment, increasing their abundance and biodiversity as well as biological activity (Adugna 2016; Kim et al. 2015; Pane et al. 2015). Compost properties make it suitable for reclamation of heavy metal contaminated areas, however, it must be sanitary safe and free from additional pollutant loads.

Effective methods of restoring soil homeostasis are still sought due to the progressive contamination of the soil environment by heavy metals. This was a prerequisite for this research, which primary purpose was to assess the suitability of stabilized compost in restoring microbial and biochemical balance of the soil.

## Material and Methods

### Soil Material

Loamy sand from the Didactic and Experimental Station in Tomaszkowo (north-eastern Poland), located in the Olsztyn Lakeland, was used for the experiment. This area is dominated by typical brown soils formed mainly on sands and clays. The material was sampled from the topsoil layer (0–20 cm), which was classified as Eutric Cambisols (World Reference Base of Soil Resources 2014). Complete characterization of soil material is shown in Table 1

**Table 1:** Selected physicochemical properties of the soil

Parameter	Unit	Value
Soil texture		
< 0.002	%	1.49
0.002–0.05		17.98
0.05–2.00		80.56
pH	–	5.60
Total organic carbon	g kg^−1^ DM of soil	10.00
Total nitrogen		0.58
Available cations		
P	mg kg^−1^ DM of soil	96.32
K		179.08
Mg		50.17
Exchangeable cations		
K^+^		217.73
Ca^2+^	mg kg^−1^ DM of soil	568.60
Na^+^		100.34
Mg^2+^		64.52
Hydrolytic acidity (HAC)		18.66
Total exchangeable bases (TEB)	mM(+) kg^−1^ DM of soil	40.00
Cation exchange capacity (CEC)		58.66
Base saturation (BS)	%	68.19
Total zinc		22.68
Available zinc	mg kg^−1^ DM of soil	9.13

### Compost Characteristics

The compost used in the experiment was produced by Nolet (Poland). It was produced by aerobic composting of coniferous sawdust and turkey litter. It was characterized by the following parameters: pH (in 1 M KCl)—7.08; organic carbon content—402.0 g kg^−1^; total nitrogen content—23.1 g kg^−1^; cations in the assimilable form (g kg^−1^): P—4.7, K—10.2, Mg—2.2; exchangeable cations (g kg^−1^): K^+^—0.8, Ca^2+^—2.7; Na^+^—0.3; and Mg^2+^—0.5.

### Experimental Conditions

The introduction of heavy metals into the soil can lead to its degradation by reducing the nutrients necessary for the growth and development of living organisms, mainly microorganisms. Replenishment of nutrient deficiencies, especially in degraded areas, is essential for the proper functioning of soil ecosystems. Therefore, the study was conducted to determine the effect of differentiated doses of zinc and compost on soil biological properties. Model studies were carried out under strictly controlled conditions in order to avoid the influence of other factors on the microbiological and biochemical properties of the soil. In 150 cm^3^ glass beakers, 100 g of air dry mass of soil material was added, into which zinc and compost were put in appropriate objects. Zinc was applied as an aqueous solution of ZnCl_2_ at a dose of: 0, 250, 500, 750, 1000, and 1250 mg Zn^2+^ kg^−1^ DM of soil. Compost calculated based on organic carbon content was used in the amount corresponding to the level of 0, 10, and 20 g C_org_ kg^−1^ DM of soil. In the experiment, 108 glass beakers were prepared, i.e., 6 beakers with different doses of zinc × 3 doses of compost, and it means 18 combinations in 3 replications (54 in total). Samples were prepared separately for two study terms (54 samples × 2 terms, it means 108 experimental beakers). After thorough homogenizing of soil samples, they were incubated at 25 °C for 2 and 20 weeks. Throughout the experiment, the moisture content of soil samples was maintained at 50% of the capillary water capacity.

### Microbiological, Biochemical and Physicochemical Analyses of the Soil

In week 2 and 20 of the incubation period, the number of the following groups of microorganisms was determined: organotrophic bacteria on Bunt and Rovira (1955) soil extracts, oligotrophic and copiotrophic bacteria—medium with peptone and meat extract, according to Oht and Hattori (1983), actinomycetes—Küster and Williams medium with nystatin and actidione antibiotics, according to Parkinson et al. (1971), and fungi—on glucose-peptone agar with Bengal rose and aureomycin according to Martin (1950). The ecophysiological diversification index (EP) was determined based on the abundance of microorganisms (organotrophic bacteria, actinomycetes, fungi), on the basis of which the diversity of the soil environment was inferred (De Leij et al. 1993). Colony growth observations were performed at 1 day intervals for a period of 8 days. The enzymatic activities of dehydrogenases (Öhlinger 1996), catalase and urease (Alef and Nannipieri 1998) and acid phosphatase, alkaline phosphatase, and β-glucosidase (Alef et al. 1998) were also determined in week 2 and 20 of soil incubation. A detailed procedure for determining the activity of the enzymes tested was described in the work of Borowik et al. (2014). The biostimulation index (IF_b_) was also calculated, on the basis of which the effect of compost on microbial multiplication and soil enzyme activity was evaluated, according to the formula provided by Kaczyńska et al. (2015):

$$ {\mathrm{IF}}_{\mathrm{b}}=\frac{A_{\mathrm{b}}}{A} $$

where *A*_b_—enzyme activity or number of microorganisms in soil with compost, *A*—enzyme activity or number of soil microorganisms without added compost.

Soil physicochemical analyses were carried out in week 20 of soil incubation, which included the determination of pH in 1 mol dm^−3^ KCl, hydrolytic acidity, sum of exchangeable alkaline cations, organic carbon content, and total nitrogen content and exchangeable cations (K^+^, Na^+^, Ca^2+^, Mg^2+^), according to methods described in Harris’s work (2006). Soil microbiological, biochemical, and physicochemical analyses were performed in three replicates.

### Statistical Analysis

Based on the results obtained, statistical analysis was performed using the Statistica 12 (StatSoft, Inc. 2014) software package. Statistical analysis included percentage involvement of factors of the observed diversity—*η* ^2^; principal component analysis (PCA) of microbial counts and enzyme activities; homogeneous groups determination by the Tukey’s honestly significant difference (HSD) test using ANOVA (at *P* < 0.01); correlation matrix between microbiological, biochemical, and physicochemical properties of the soil.

## Results and Discussion

### Soil Microbiome

**Fig. 1:**
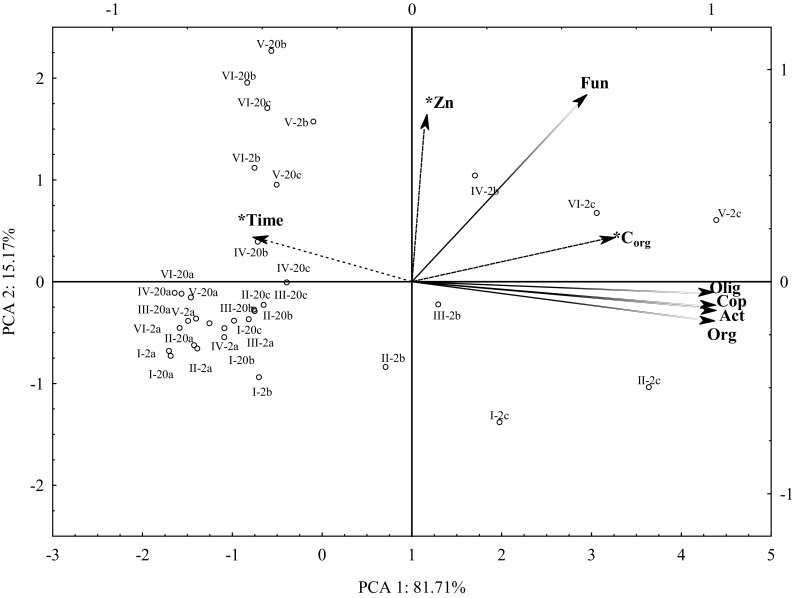
The count of selected groups of microorganism in zinc-contaminated soil supplemented with compost (PCA analysis). Org organotrophic bacteria, Cop copiotrophic bacteria, Olig oligotrophic bacteria, Act actinomycetes, Fun fungi; dose of zinc (mg Zn^2+^ kg^−1^ DM soil): I—0, II—250, III—500, IV—750, V—1000, VI—1250; dose of C_org_ (g kg^−1^ DM soil): a—0, b—10, c—20; incubation time: 2—two weeks, 20—twenty weeks

The soil environment is a very complex structure, but at the same time, it is a natural storage of diverse substances, including heavy metals, which can lead to unsustainable soil ecosystems. Zinc is one of these elements, which on the one hand is present in the soil environment as a microelement and plays a vital role in the proper functioning of living organisms. On the other hand, when it occurs in the soil in excessive amounts, it can become toxic, leading to the disruption of its biological and chemical balance (Pardo et al. 2014; Soares et al. 2015). On the basis of the results of the present study, it can be concluded that all analyzed factors (zinc dose, compost fertilization, and soil incubation time) significantly influenced microbiological properties of the soil (Table 2). Compost soil fertilization most highly affected the count of organotrophic bacteria, oligotrophic bacteria, and actinomycetes, zinc dosage—fungi count, while soil incubation time—the number of copiotrophic bacteria. Principal component analysis also confirmed these dependencies (Fig. 1). It was found that two basic groups were formed. The first group consisted of vectors representing the count of organotrophic bacteria, copiotrophic bacteria, oligotrophic bacteria, and actinomycetes. This group was positively correlated with the dose of zinc and compost, and negatively with soil incubation time. The second group was formed by a vector representing fungi that positively correlated with the dose of zinc and compost and soil incubation time. The positioning of the vectors representing the variables suggested that microorganisms forming the first group (organotrophic bacteria, copiotrophic bacteria, oligotrophic bacteria, and actinomycetes) reacted similarly to the factors used in the experiment. Considering the distribution of the cases, it was observed that zinc effect on soil microorganisms varied over time. At week 2 of the treatment, zinc at doses from 250 to 1250 mg Zn^2+^ kg^−1^ contributed to growth stimulation of copiotrophic bacteria, oligotrophic bacteria, and fungi. In the case of actinomycetes, a decrease in their number was recorded following the introduction of zinc at 1000 and 1250 mg Zn^2+^ kg^−1^. Organotrophic bacteria also responded to zinc reduction with a lower count, but after its administration at a dose of 1250 mg Zn^2+^ kg^−1^. In the 20th week of soil incubation, the count of oligotrophic bacteria and fungi increased after zinc introduction into the soil at all tested doses, identically to week 2. At the same time point, zinc doses of 1000 and 1250 mg Zn^2+^ kg^−1^ inhibited the growth of copiotrophic bacteria and actinomycetes, and doses of 500 to 1250 mg Zn^2+^ kg^−1^ inhibited the multiplication of organotrophic bacteria. It was also observed that the count of organotrophic bacteria, copiotrophic bacteria, oligotrophic bacteria, and actinomycetes was higher at week 2, and that of fungi at week 20. This is indicated by the opposite arrangement of the vectors describing these groups relative to the variable vector representing soil incubation time. The excessive amounts of heavy metals may result in the reduction of the soil microbiome pool. In many works (Pérez-de-Mora et al. 2006; Wang et al. 2007; Zhou et al. 2017), microbial biomass was reduced due to heavy metal action. In turn, Sang-Hwan et al. (2009) noted the negative impact of these elements on soil respiration.

**Table 2:** Percentage proportion of the observed variability factors *η* ^2^

Factors	Parameters										
	Org	Cop	Olig	Act	Fun	Deh	Cat	Pac	Pal	Ure	Glu
Dose of Zn	5.47	3.00	11.03	4.38	40.34	17.10	33.47	65.14	7.72	0.21	16.32
Dose of C_org_	37.91	27.96	42.88	39.26	32.52	29.07	50.93	25.13	56.23	58.54	56.38
Incubation time	23.59	35.45	17.02	23.76	1.21	20.80	3.82	0.11	10.84	16.96	6.00
Dose of Zn × dose of C_org_	2.38	1.25	6.50	1.41	13.30	5.23	6.09	2.09	7.95	0.45	14.13
Dose of Zn × incubation time	2.19	2.53	2.58	2.73	4.76	5.50	1.64	3.01	2.62	5.58	2.58
Dose of C_org_ × incubation time	25.73	26.56	15.91	24.27	3.48	18.82	2.88	1.83	12.32	12.98	0.98
Dose of Zn × dose of C_org_ × incubation time	1.54	1.32	3.26	1.97	3.13	3.48	1.01	2.66	2.32	5.17	3.60
Error	1.18	1.93	0.82	2.22	1.24	0.01	0.16	0.04	0.00	0.11	0.01

*Zn* zinc, *Org* organotrophic bacteria, *Cop* copiotrophic bacteria, *Olig* oligotrophic bacteria, *Act* actinomycetes, *Fun* fungi, *Deh* dehydrogenases, *Cat* catalase, *Pac* acid phosphatase, *Pal* alkaline phosphatase, *Ure* urease, *Glu* β-glucosidase

Increasing emphasis is being placed on the reclamation of soils under heavy metal pressure due to the need of sustaining proper condition of the soil environment. Soil remediation techniques are based primarily on two processes: extraction and stabilization of soil contaminants. Extraction is a costly process, thus it is better to use “in situ” stabilization techniques that require less financial effort and can be applied in larger areas contaminated with heavy metals (Pérez de Mora et al. 2005; Lee et al. 2009). According to Baker et al. (2011), organic matter content can be of great importance in maintaining good microbiological condition of the soil subjected to heavy metal pressure. In own research, the introduction of compost into the soil was highly stimulating on soil microbiome, as evidenced by the arrangement of vectors in particular cases. Both in the 2nd and 20th week of incubation, the highest counts of copiotrophic bacteria, oligotrophic bacteria, actinomycetes, and fungi were recorded in objects with the highest compost dosage (20 g C_org_ kg^−1^ DM of soil). A slightly different relationship to soil compost was observed for organotrophic bacteria. Their growth in the 2nd week was the highest in soil with the compost amount of 20 g C_org_ kg^−1^ DM of soil, while at week 20, the number of these bacteria remained at a similar level after the introduction of compost at 10 and 20 g C_org_ kg^−1^ DM of soil. Soil microbiome changes largely depend on the state of the environment and the dose of compost used (Baker et al. 2011). Nevertheless, the dynamics of changes in soil is the greatest in the initial period after compost application, as evidenced by increased CO_2_ emission from the soil, which gradually decreases over time (Debosz et al. 2002). Compost added to the soil environment primarily increases the pool of microorganisms, which is closely associated with soil processes triggered by the microbiome (Chang et al. 2007). Stimulation of autochthonous microorganisms, as a result of introducing nutrients contained in compost, improves the biochemical properties of the soil environment (Marinari et al. 2000). In addition, the efficiency of microorganisms in the metabolism of organic carbon sources is higher and the colonization of plant roots by fungi increases (Civeira 2010). The strongly degraded soils are deprived of organic matter; hence, the addition of compost leads to an improvement of general soil condition, through supplementing the carbon sources and other nutrients (Pane et al. 2015). In addition, organic matter plays a fundamental role in the proper functioning of soil ecosystems and determines soil fertility and its resistance to environmental degradation factors (Civeira 2010). The study of Baker et al. (2011) showed that the soil after compost application was dominated by bacteria. In turn, based on the analysis of fatty acids, Bastida et al. (2008) observed an increase in the ratio of G^+^ to G^−^ bacteria after compost application to the soil. In addition, similar changes were observed with respect to mycorrhizal fungi in relation to total fungi number. The biostimulation index (IF_b_) shown in Table 3 confirmed the beneficial effect of compost on the development of soil microorganisms. Mean IF_b_ index values calculated in all zinc doses and experimental time points indicated that soil supplementation with compost had the most favorable impact on the development of actinomycetes (IF_b_ = 10.78). It was also observed that the positive effect of compost on the microbiological properties of the soil was more effective when introduced at a dose 20 g C_org_ kg^−1^ DM of soil. Vinhal-Freitas et al. (2010) also observed the stimulating effect of compost applied at doses of 10 and 20 g kg^−1^ of soil on the propagation of microorganisms. Taking into account soil incubation time, it was noted that biostimulation index values were higher at week 2 than at week 20. This higher stimulating effect of compost in the second week of soil incubation resulted from the supply of the proper amount of fresh organic matter to the soil, which provided nutrients for microorganisms. In time, organic matter was mineralized, resulting in fewer nutrients needed for the proliferation of microorganisms at week 20 (Adugna 2016). In the 20th incubation week, the introduction of compost at a dose of 10 mg C_org_ kg^−1^ DM of soil. Negatively affected the proliferation of the copiotrophic bacteria, because IF_b_ index values in objects with zinc dosages from 500 to 1250 mg Zn^2+^ kg^−1^ DM of soil were lower than 1.

**Table 3:** Effect of compost on the abundance of soil microorganisms expressed by the biostimulation index (IF_b_)

Dose of zinc (mg Zn^2+^ kg^−1^)	Org		Cop		Olig		Act		Fun	
	Incubation time (weeks)									
	2	20	2	20	2	20	2	20	2	20
10 g C_org_ kg^−1^ DM of soil										
0	3.61c,d	1.79a	4.88a,b,c	1.21a	5.85c,d,e	5.53a,b	4.56c	3.42a,b,c,c	1.20b	10.22a,b
250	3.71c,d	2.45a	4.06b,c	1.01a	6.37c,d	1.80b	4.39c	2.04b,c	2.97a,b	3.16c,d,e
500	2.26c,d	3.48a	3.79b,c	0.79a	6.06c,d	2.66a,b	4.60c	1.35c	2.76a,b	1.56c
750	5.33b,c,d	3.21a	6.89a,b,c	0.73a	2.25d,e	3.84a,b	15.81a,b,c	1.00c	6.33a,b	2.16c
1000	2.91c,d	2.30a	2.88c	0.96a	2.38d,e	2.39a,b	7.65b,c	2.42a,b,c	7.06a,b	4.99b,c
1250	1.60d	3.14a	4.02b,c	0.85a	1.72e	1.81b	2.33c	2.69a,b,c	6.84a,b	4.50b,c
Average	3.24	2.73	4.42	0.93	4.11	3.01	6.56	2.15	4.53	4.43
*r*	− 0.38	0.49	− 0.18	− 0.62	− 0.89	− 0.58	0.11	− 0.17	0.94	− 0.38
20 g C_org_ kg^−1^ DM of soil										
0	9.99a,b,c	1.90a	14.87a,b	1.47a	11.60a,b	4.95a,b	26.85a,b,c	4.72a,b,c,c	5.79a,b	16.22a
250	8.42a,b,c,d	1.99a	9.45a,b,c	1.21a	7.60a,b,c	1.89b	13.25a,b,c	2.75a,b,c	6.81a,b	3.59c,d,e
500	7.36a,b,c,d	3.43a	8.85a,b,c	1.12a	13.17a	1.99b	9.24b,c	3.10a,b,c	4.26a,b	1.78c
750	14.06a	3.04a	14.52a,b,c	1.25a	5.32c,d,e	6.34a	32.65a,b,c	2.04b,c	6.64a,b	1.58c
1000	11.85a,b	2.74a	16.04a	1.51a	9.05a,b,c	4.59a,b	51.38a	6.41a,b	7.92a	3.00d,e
1250	8.27a,b,c,d	3.36a	16.45a	1.13a	9.00a,bc,	3.96a,b	46.83a,b	7.22a	8.69a	4.18b,c
Average	9.99	2.74	13.36	1.28	9.29	3.95	30.03	4.37	6.69	5.06
*r*	0.18	0.73	0.53	− 0.22	− 0.31	0.23	0.74	0.57	0.69	− 0.60

Homogeneous groups were marked with the same letters separately for each group of microorganisms and soil incubation time (two-way ANOVA performed using Tukey’s *T* test at *P* < 0.01)

*Org* organotrophic bacteria, *Cop* copiotrophic bacteria, *Olig* oligotrophic bacteria, *Act* actinomycetes, *Fun* fungi, *r* Pearson’s linear correlation coefficient

**Fig. 2:**
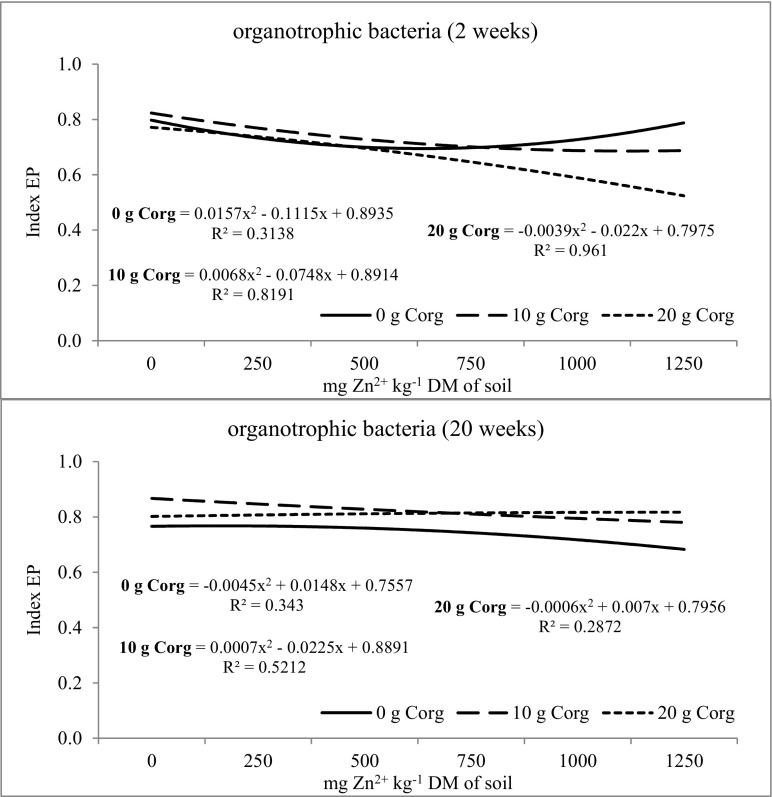
The ecophysiological diversification index (EP) of organotrophic bacteria in zinc-contaminated soil supplemented with compost. 0 g C_org_—soil without compost addition; 10 g C_org_—soil with 10 g compost calculated based on the organic carbon content; 20 g C_org_—soil with 20 g compost calculated based on the organic carbon content

**Fig. 3:**
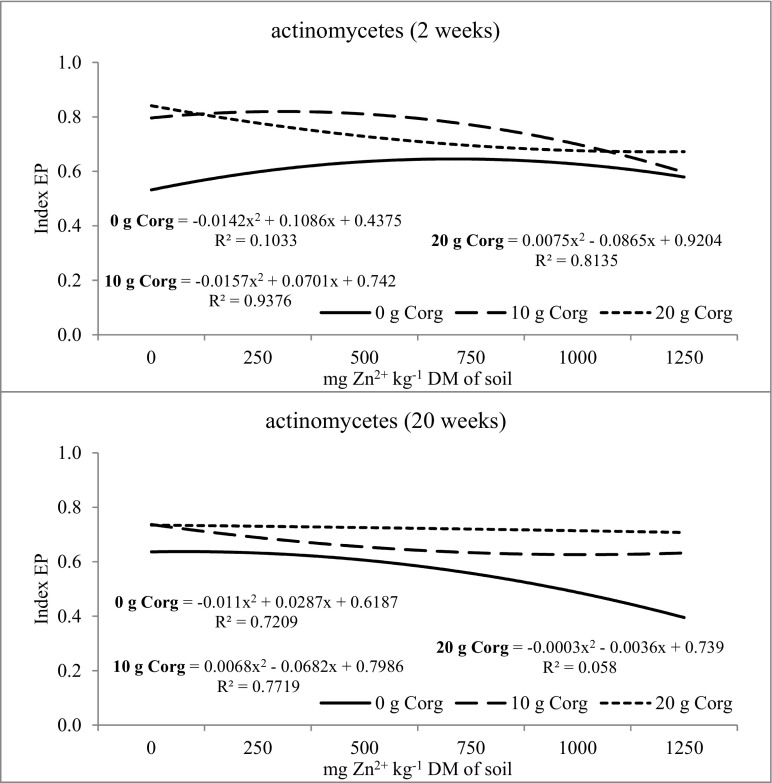
The ecophysiological diversification index (EP) of actinomycetes in zinc-contaminated soil supplemented with compost. 0 g C_org_—soil without compost addition; 10 g C_org_—soil with 10 g compost calculated based on the organic carbon content; 20 g C_org_—soil with 20 g compost calculated based on the organic carbon content

**Fig. 4:**
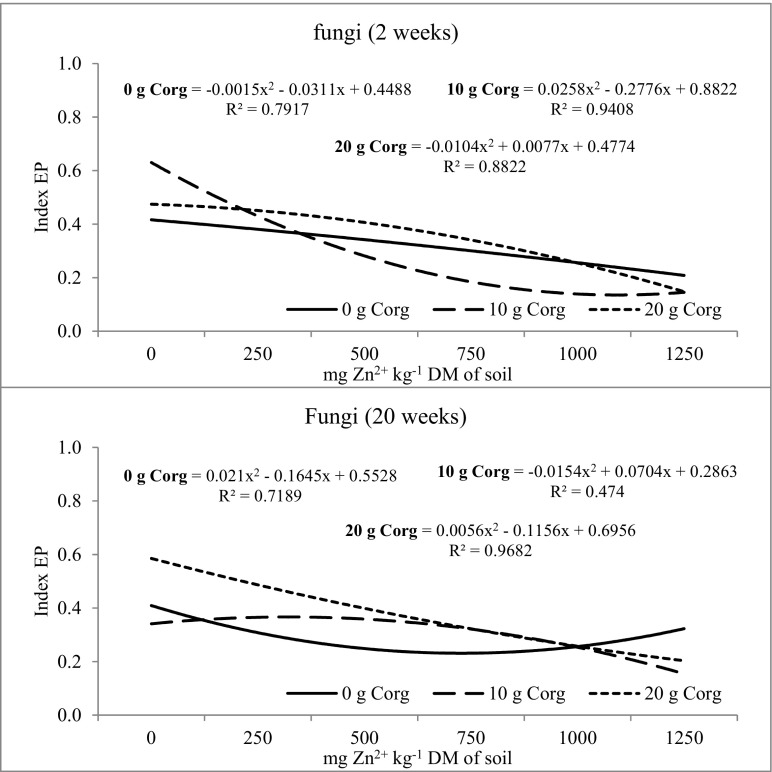
The ecophysiological diversification index (EP) of fungi in zinc-contaminated soil supplemented with compost. 0 g C_org_—soil without compost addition; 10 g C_org_—soil with 10 g compost calculated based on the organic carbon content; 20 g C_org_—soil with 20 g compost calculated based on the organic carbon content

Biodiversity of microorganisms determined by the soil ecophysiological diversification index (EP) was also modified by the investigated factors (Figs. 2, 3, and 4). The value of the EP index of organotrophic bacteria at week 2 and 20 of the experiment was similar (Fig. 2). It was recorded that the mean EP index value at week 2 and 20 of soil incubation was 0.740. Zinc dose did not significantly influence the ecophysiological diversification index of organotrophic bacteria, while at week 2, the largest changes were caused by the dose of 250 mg of Zn^2+^ kg^−1^ and at week 20—by the dose of 1250 mg of Zn^2+^ kg^−1^. In the 2nd week of soil incubation, compost introduction at a dose of 10 and 20 g C_org_ kg^−1^ DM of soil reduced the diversity of organotrophic bacteria. Although the biodiversity of organotrophic bacteria was reduced in the 2nd week after compost application, its stimulating effects on the multiplication of these bacteria was noted. The introduction of compost has increased the amount of available carbon, thereby increasing the number of organotrophic bacteria. However, at week 20, the increase of the EP index value in the objects with composts was already observed, indicating an increase in the biodiversity of these bacteria. The diversity of actinobacteria was altered by the tested factors (Fig. 3). The EP value was observed to increase in the second week of soil incubation upon zinc application to the soil at doses from 250 to 750 mg Zn^2+^ kg^−1^, while at week 20—upon zinc doses of 250 and 500 mg Zn^2+^ kg^−1^. The prolonged retention of high zinc doses in non-fertilized objects resulted in decreased biodiversity of actinomycetes. The introduction of compost into the soil contributed to the increase of actinomycetes biodiversity. This was particularly evident at week 20 of the study in soil samples, to which compost was added at 10 and 20 g C_org_ kg^−1^ DM of soil. The ecophysiological diversification index was altered by the zinc and compost dosage and soil incubation time (Fig. 4). Zinc caused a decrease in the EP index when present in excessive amounts in the soil. In week 2, the dose of 1000 mg of Zn^2+^ kg^−1^ caused the largest changes, while 750 mg of Zn^2+^ kg^−1^ in week 20. Compost introduction to the soil exerted a varied effect on the EP index value in fungi. At week 2, the EP index was at a similar level in objects both with and without compost. At week 20, a positive effect of compost on the increase of fungal biodiversity was observed. Increased biodiversity of the soil microbiome under compost influence could indicate its protective role against the action of heavy metals, including zinc. Compost supplementation can also affect microbiome by promoting microorganisms that are more resistant to heavy metals in the soil. It can be stated that compost fulfills its role in stabilizing the microbial diversity of soil contaminated with zinc.

### Soil Enzymes

**Fig. 5:**
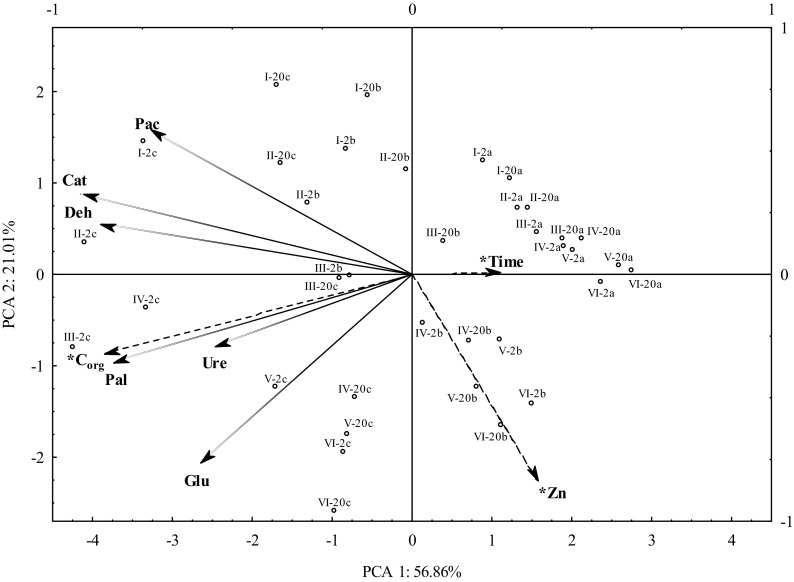
The activity of selected enzymes in zinc-contaminated soil supplemented with compost (PCA analysis). Deh dehydrogenases, Cat catalase, Ure urease, Pac acid phosphatase, Pal alkaline phosphatase, Glu β-glucosidase; dose of zinc (mg Zn^2+^ kg^−1^ DM soil): I—0, II—250, III—500, IV—750, V—1000, VI—1250; dose of C_org_ (g kg^−1^ DM soil): a—0, b—10, c—20; incubation time: 2—two weeks, 20—twenty weeks

The investigated factors (zinc dose, compost dose, and soil incubation time) also had a significant effect on soil biochemical properties (Table 2). Acid phosphatase activity was also primarily affected by zinc dose (in 65.14%). In turn, compost dose affected dehydrogenase activities in 29.07%, catalase—in 50.93%; urease—in 58.54%; alkaline phosphatase—in 56.23; and β-glucosidase—in 56.38%. Enzymatic activity time was the least affected by soil incubation, from 0.11% (acid phosphatase activity) to 20.80% (dehydrogenase activities). Results of principal component analysis, shown in Fig. 5, revealed that zinc caused significant changes in the enzymatic activity of the soil, but its activity changed over time. Zinc applied to the soil at doses of 250 to 1250 mg Zn^2+^ kg^−1^ was a potent inhibitor of the activity of dehydrogenases, catalase, and acid phosphatase. Based on the literature data (Borowik et al. 2014; Chaperon and Sauvé 2007; Kucharski et al. 2011; Strachel et al. 2017a, dehydrogenases are the most sensitive enzymes to excessive amounts of zinc in the environment. According to Yang et al. (2006), catalase also shows high sensitivity to zinc. In the current study, urease activity increased with the introduction of 250 and 500 mg of Zn^2+^ kg^−1^ to the soil in week 2 and from 250 to 750 mg Zn^2+^ kg^−1^ in week 20. The inverse relationship was recorded in the case of β-glucosidase. Alkaline phosphatase activity decreased after zinc addition in doses from 500 to 1250 mg Zn^2+^ kg^−1^. Kucharski et al. (2011) and Borowik et al. (2014) also observed negative effects of zinc on soil enzymatic activity, especially when applied at contaminating doses. They also noted that this inhibitory effect was long lasting. Moreno et al. (2009) reported that the activity of soil enzymes, i.e., urease, acid phosphatase, and β-glucosidase, decreased with the dose of this element in zinc-contaminated soil. The decrease in β-glucosidase activity in zinc-stressed soil was noted by, among others, Castaldi et al. (2004), Hinojosa et al. (2004), Kunito et al. (2001), and Moreno et al. (2009). Studies of other authors also confirmed that the activity of soil enzymes, i.e., dehydrogenases (Pérez-de-Mora et al. 2006; Sang-Hwan et al. 2009), phosphatases (Pérez-de-Mora et al. 2006; Wang et al. 2007), urease (Sang-Hwan et al. 2009), β-glucosidase, and arylsulfatase (Pérez-de-Mora et al. 2006), could be affected by heavy metals. In this study, the introduction of compost into the soil contributed positively to the activity of the analyzed soil enzymes. However, higher neutralizing effects of compost on the biochemical properties of zinc-contaminated soil were visible in objects with doses of 20 g C_org_ kg^−1^ DM of soil. Compost addition to the soil contaminated with zinc doses from 250 to 1250 mg Zn^2+^ kg^−1^ contributed to the increase in β-glucosidase activity. Moreno et al. (2009) believed that organic matter introduction into the soil reduced the negative effects of zinc on soil enzymes and even stimulated their activity. This stimulating effect of compost on enzymes indicates the strong influence of organic matter on the increase of microbiome abundance, and thus the improvement of soil biochemical properties. Enzyme secretion by microorganisms is increased as a result of microbial proliferation and development (Babalola et al. 2012; Civeira 2010). The increase in enzyme activity may also result from zinc binding by organic matter, which occurs through the adsorption and formation of chelated bonds with complex properties (Jiao et al. 2011; Wyszkowska et al. 2015). In addition, the highest activity was observed in the objects in the 2nd week of soil incubation, both without compost and with its addition. Soil biostimulation indices (IF_b_) showed that compost not only alleviated the negative effects of zinc, but also activated soil enzymes (Table 4). According to mean IF_b_ values, calculated based on all zinc doses and experimental time points, compost had the greatest stimulating effect on soil enzyme activities in the following order (from the highest to the lowest): urease (22.79), dehydrogenases (19.51), alkaline phosphatase (13.96), catalase (2.79), β-glucosidase (2.50), and acid phosphatase (1.52). Compost exerted a more stimulating effect on dehydrogenases, catalase, and alkaline phosphatase activities at week 2 of soil incubation, while on urease, acid phosphatase, and β-glucosidase activities at week 20. As in the case of soil microorganisms, the activity of soil enzymes was considerably higher in objects with compost dose of 20 g C_org_ kg^−1^ DM of soil. Study conducted by Vinhal-Freitas et al. (2010) confirmed the beneficial effect of compost on soil enzymatic activity. It was recorded that compost introduced into the soil, particularly at a dose of 20 g kg^−1^ DM of soil, significantly increased the activity of β-glucosidase, acid phosphatase, and alkaline phosphatase when compared to the variant without organic matter addition. After 20 weeks of incubation, zinc contamination significantly affected microbiological, biochemical, and physicochemical properties of the soil. Table 5 shows the correlation coefficients between the variables tested and soil parameters. There was a highly significant negative correlation observed between zinc dose and the abundance of organotrophic bacteria, copiotrophic bacteria and actinomycetes, and dehydrogenase and catalase activities. A significantly positive correlation was noted between zinc dose and fungal count and β-glucosidase activity. The zinc dose also significantly negatively correlated with the physicochemical properties of the soil, except for soil hydrolytic acidity. These changes in environmental conditions also significantly correlated with the results obtained. The number of microorganisms and soil enzymes considerably decreased with the increase in soil environment acidity, with the exception of fungi and β-glucosidase. The introduction of compost into the soil significantly modified the physicochemical properties of the soil environment. Compost is a rich source of biogenes, i.e., C, N, and P, thus, it increases the pool of available nutrients (Baker et al. 2011; Farrell and Jones 2010). The type of substance from which compost is produced largely determines its properties. Many studies (Baker et al. 2011; Farrell and Jones 2010; Pérez de Mora et al. 2005; Pérez-de-Mora et al. 2006; Zhou et al. 2017) reported an increase in the pH value after compost application to the soil, which reduced the bioavailability of trace elements. The magnitude of the effect can be determined by the amount of compost introduced. The higher the compost dose, the more limited zinc bioavailability (Taiwo et al. 2016). The study of Paradelo et al. (2011) analyzed in detailed metal chelates and found that organic matter could play a key role in the immobilization of trace elements by forming compounds with them. Moreover, the modification of soil physicochemical properties caused changes in soil microbiome. In the study of Pardo et al. (2014), the activity of soil enzymes (cellulase, β-glucosidase, urease) was positively correlated with the pH and availability of biogenic elements (C, N, P, K), whereas negatively correlated with heavy metal content. Compost addition, by increasing organic matter content, stabilizes the microbial processes in the soil by changing the C/N ratio (Taiwo et al. 2016). Many authors pointed to the increase of microbial C biomass after compost application (Baker et al. 2011; Bastida et al. 2008; Pérez de Mora et al. 2005; Pérez-de-Mora et al. 2006; Zhou et al. 2017). According to Chang et al. (2007), soil respiration, nitrification rate and stimulation of enzymatic activity (dehydrogenases, cellulases, β-glucosidases, urease, arylsulfase, acid phosphatase, and alkaline phosphatase)are also elevated with increasing microbial C biomass. These effects were increased with the applied dose of compost. There was a positive effect of compost on enzymes, i.e., dehydrogenases (Bastida et al. 2008; Pérez de Mora et al. 2005), acid phosphatase (Baker et al. 2011), alkaline phosphatase (Baker et al. 2011; Bastida et al. 2008), arylsulfase (Baker et al. 2011; Pérez de Mora et al. 2005), urease (Bastida et al. 2008), β-glucosidase (Baker et al. 2011; Bastida et al. 2008; Debosz et al. 2002; Pérez de Mora et al. 2005); and FDA (Debosz et al. 2002) hydrolytic activity. Moreover, it was observed that the positive effects of soil composting can persist for a long time—19 months (Bastida et al. 2008). In turn, Baker et al. (2011) noted the positive effect of compost on enzyme activity even after 711 days. This allows concluding that compost is a material that has a long-term positive effect on the microbiological, biochemical, and physicochemical properties of the soil, thereby reducing the negative effects of soil contamination with zinc. In the present study, compost showed a positive effect on microbial and biochemical properties of the soil already in the second week of the experiment, and these effects persisted throughout the experiment.

**Table 4:** Effect of compost on the abundance of soil enzymes expressed by the biostimulation index (IF_b_)

Dose of zinc (mg Zn^2+^ kg^−1^)	Deh		Cat		Ure		Pac		Pal		Glu	
	Incubation time of soil (weeks)											
	2	20	2	20	2	20	2	20	2	20	2	20
10 g C_org_ kg^−1^ DM of soil												
0	3.41d	3.02c,d	2.08e,f	2.10e	8.89c,d	9.26b	1.05e	1.48f,g,h	3.77d	2.28g	1.42h	1.12i
250	6.02c,d	2.36c,d	2.61c,d,e	1.97e	7.86d	10.83b	1.35c	1.42g,h	6.81d	4.68e,f,g	1.75g	1.03i
500	12.52c,d	1.90d	2.38e	2.14d,e	5.35d	9.95b	1.15d	1.52f,g,h	13.74c	6.49d,e	1.72g	1.55g,h
750	24.57c,d	3.38c,d	2.48d,e	1.87e	5.33d	10.30b	1.07e	1.19i	15.53c	7.23d,e	1.94f	2.59f
1000	7.09c,d	4.42b,c	1.71e,f	1.93e	4.11d	23.56b	1.05e	1.81c,d	5.03d	7.79d	2.20e	3.75d
1250	7.84c,d	4.32b,c	1.17f	2.05e	4.50d	39.00b	1.11d,e	1.63d,e,f	4.12d	6.61d,e	2.61c	4.79b
Average	10.23	3.23	2.07	2.01	6.01	17.150	1.13	1.51	8.17	5.85	1.94	2.47
*r*	0.26	0.74	− 0.70	− 0.34	− 0.92	0.834	− 0.32	0.40	− 0.02	0.83	0.96	0.96
20 g C_org_ kg^−1^ DM of soil												
0	6.24	3.30c,d	3.76a,b,c	3.07b,c	34.56a	28.13b	1.12d,e	1.60e,f,g	7.74d	3.63f,g	1.91f	1.09i
250	13.15c,d	2.74c,d	3.89a,b	2.72c	30.89a	31.25b	1.43b	1.91c	14.75c	5.59d,e,f	2.24e	1.45h
500	30.18c	4.30b,c	3.78a,b	2.63c,d	17.40b	31.85b	1.46b	1.75c,d,e	35.43a,b	13.75c	2.62b,c	1.63g
750	111.72a	5.78b	4.77a	3.01b,c	14.89b,c	26.12b	1.66a	1.35h,i	39.18a	17.40b	2.51d	2.99e
1000	82.19b	6.21a,b	3.92a,b	3.48a,b	10.28c,d	55.33b	1.29c	2.37b	38.76a	21.54a	2.70b	4.31c
1250	113.44a	8.05a	3.58b,c,d	3.86a	7.67d	119.33a	1.73a	2.86a	33.20b	19.99a,b	3.40a	6.62a
Average	59.49	5.06	3.95	3.13	19.28	48.67	1.45	1.97	28.18	13.65	2.56	3.01
*r*	0.90	0.96	0.02	0.76	− 0.96	0.77	0.67	0.70	0.80	0.95	0.93	0.94

Homogeneous groups were marked with the same letters separately for each group of enzymes and soil incubation time (two-way ANOVA performed using Tukey’s *T* test at *P* < 0.01)

*Deh* dehydrogenases, *Cat* catalase, *Ure* urease, *Pac* acid phosphatase, *Pal* alkaline phosphatase, *Glu* β-glucosidase, *r* Pearson’s linear correlation coefficient

**Table 5:** Correlation coefficients between studied variables and enzyme activities, the count of microorganisms and physicochemical properties of the soil

Variables	Zn	Org	Cop	Olig	Act	Fun	Deh	Cat	Pac	Pal	Ure	Glu	pH	HAC	TEB	CEC
Org	− 0.32*															
Cop	− 0.64**	0.38**														
Olig	− 0.08	0.74**	0.35**													
Act	− 0.49**	0.70**	0.75**	0.55**												
Fun	0.70**	0.05	− 0.53**	0.09	− 0.22											
Deh	− 0.71**	0.27	0.39**	0.10	0.46**	− 0.34*										
Cat	− 0.62**	0.65**	0.59**	0.49**	0.82**	− 0.19	0.80**									
Ure	− 0.80**	0.62**	0.66**	0.36**	0.81**	− 0.36**	0.78**	0.92**								
Pac	0.06	0.71**	0.34*	0.78**	0.70**	0.29*	0.02	0.53**	0.40**							
Pal	0.19	0.53**	0.22	0.67**	0.59**	0.46**	0.08	0.53**	0.32*	0.92**						
Glu	0.52**	0.29*	− 0.26	0.46**	0.06	0.83**	− 0.25	0.06	− 0.11	0.63**	0.76**					
pH	− 0.83**	0.50**	0.55**	0.27*	0.63**	− 0.36**	0.91**	0.87**	0.90**	0.22	0.21	− 0.16				
HAC	0.84**	− 0.61**	− 0.68**	− 0.43**	− 0.75**	0.41**	− 0.72**	− 0.85**	− 0.92**	− 0.41**	− 0.31*	0.09	− 0.90**			
TEB	− 0.40**	0.81**	0.49**	0.70**	0.84**	0.08	0.59**	0.92**	0.81**	0.77**	0.74**	0.37**	0.73**	− 0.77**		
CEC	− 0.32*	0.81**	0.44**	0.71**	0.82**	0.16	0.54**	0.89**	0.76**	0.79**	0.78**	0.43**	0.67**	− 0.70**	0.99**	
BS	− 0.49**	0.82**	0.49**	0.69**	0.79**	0.07	0.58**	0.88**	0.83**	0.73**	0.68**	0.38**	0.76**	− 0.83**	0.97**	0.96**

*Zn* zinc, *Org* organotrophic bacteria, *Cop* copiotrophic bacteria, *Olig* oligotrophic bacteria, *Act* actinomycetes, *Fun* fungi, *Deh* dehydrogenases, *Cat* catalase, *Pac* acid phosphatase, *Pal* alkaline phosphatase, *Ure* urease, *Glu* β-glucosidase, *HAC* hydrolytic acidity, *TEB* exchangeable bases, *CEC* cation exchange capacity, *BS* base saturation (*p < 0.05, **p < 0.01; *n* = 53)

## Conclusions

Soil contamination with zinc significantly modified the microbiological, biochemical, and physicochemical soil properties. The application of excessive amounts of zinc to the soil reduced the number of organotrophic bacteria, copiotrophic bacteria, oligotrophic bacteria, and actinomycetes. The stimulating effect of this metal on fungi propagation was observed. The activity of soil enzymes, important due to their involvement in the circulation of key biogenic elements, was suppressed. Compost—as a rich source of biogenes—was shown to stimulate the growth of microorganisms and to increase the microbial diversity of the soil environment. An increase in the enzymatic activity of the soil was another positive effect of compost application. Organic matter suppressed the negative effects associated with excessive doses of zinc. Intensive influence of compost was observed already after 2 weeks of its application. Nevertheless, these effects persisted throughout the experiment. The reaction of the soil environment was determined by both the zinc dose and the amount of organic matter introduced. This stimulating effect of compost on soil biological properties can be conducive to reducing toxic effects of zinc. Therefore, compost can be successfully used in the bioremediation of soils contaminated with this element.
